# Areca nut extract (ANE) inhibits the progression of hepatocellular carcinoma cells via activation of ROS production and activation of autophagy

**DOI:** 10.7150/ijms.61570

**Published:** 2021-08-09

**Authors:** Po-Li Wei, Chin-Sheng Hung, Hsuan-Hsuan Lu, Uyanga Batzorig, Chien-Yu Huang, Yu-Jia Chang

**Affiliations:** 1 Division of Colorectal Surgery, Department of Surgery, Taipei Medical University Hospital, Taipei Medical University, Taipei, Taiwan.; 2 Department of Surgery, College of Medicine, School of Medicine, Taipei Medical University, Taipei, Taiwan.; 3 Cancer Research Center and Translational Laboratory, Department of Medical Research, Taipei Medical University Hospital, Taipei Medical University, Taipei, Taiwan.; 4 Graduate Institute of Cancer Biology and Drug Discovery, Taipei Medical University, Taipei, Taiwan.; 5 Department of Internal Medicine, National Taiwan University Hospital, Taipei, Taiwan.; 6 Division of General Surgery, Department of Surgery, Shuang Ho Hospital, Taipei Medical University, Taipei, Taiwan.; 7 Division of Colon and Rectal, Department of Surgery, Shuang Ho Hospital, Taipei Medical University.; 8 Graduate Institute of Clinical Medicine, School of Medicine, College of Medicine, Taipei Medical University, Taipei, Taiwan.; 9 Cell Physiology and Molecular Image Research Center, Wan Fang Hospital, Taipei Medical University, Taipei, Taiwan.; 10 Department of Pathology, Wan Fang Hospital, Taipei Medical University, Taipei, Taiwan.

**Keywords:** hepatocellular carcinoma, ANE, apoptosis, ROS, autophagy, lysosome

## Abstract

Hepatocellular carcinoma (HCC) is a worldwide health problem. Currently, there is no effective therapeutic strategy for HCC patients. Chewing areca nut is closely associated with oral cancer and liver cirrhosis. The therapeutic effect of areca nut extract (ANE) on HCC is unknown. Our results revealed that ANE treatment caused a reduction in cell viability and an increase in cell apoptosis and suppressed tumor progression in xenograft models. ANE-treated didn't induce liver tumor in nude mice. For mechanism dissection, ANE treatment caused ROS-mediated autophagy and lysosome formation. Pretreatment with an ROS inhibitor, aminoguanidine hemisulfate (AGH), abolished ANE-induced ROS production. ANE treated cells caused an increase in light chain 3 (LC3)-I to -II conversion, anti-thymocyte globulin 5+12 (ATG5+12), and beclin levels, and apoptosis related-protein changes (an increases in BAX, cleaved poly(ADP-ribose) polymerase (c-PARP), and a decrease in the Bcl-2 level). In conclusion, our study demonstrated that the ANE may be a new potential compound for HCC therapy.

## Introduction

Liver cancer, as the third leading cause of all cancer-related deaths worldwide, is responsible for about 746,000 deaths (9.1%) 1, 2. Hepatocellular carcinoma (HCC) is most prevalent in East and Southeast Asian and African countries 1, 2. The overall 5-year survival rate is still low at around 5%~10%, due to it being highly resistant to current therapeutic strategies 3. Currently, multi-kinase inhibitors, such as sorafenib and sutent, have been approved by US Food and Drug Administration for advanced HCC patients to extend the patients' lifespan by approximately 2~3 months 3. Moreover, they are associated with serious adverse side effects, and drug resistance often develops. Most patients with advanced HCC cannot tolerate surgery and do not respond to many chemotherapies, so we urgently need to find new treatments for HCC patient therapy.

Areca catechu (family Arecaceae) is widely distributed in South and Southeast Asia, and its fruit (betel quid/nut) is popularly chewed for psychoactive effects and is also known to treat ailments like parasitic diseases, dyspepsia, abdominal distension, abdominal pain, diarrhea, edema, jaundice, and many others 4, 5. In addition, areca nut extract (ANE) was demonstrated to have various pharmacological effects which range from being beneficial to those that pose serious health risks 5. The areca nut, the main component of betel quid, is considered to be a major etiologic factor of oral cancer, as the ANE contains mainly tannin and areca alkaloids (such as arecoline and arecaidine) that are potential carcinogens 6, 7. When person chews betel quid, areca nut-derived nitrosamines may methylate and cyanoethylate liver DNA 8, and it is genotoxic to hepatocytes, and thus cause liver cancer 9. The several studies reported that increased usage of betel quid is associated with the increased risk of HCC 10-12. Despite these observations, the ANE was also shown to exhibit cytotoxic effects and affects wound-healing colony formation in oral cancer cells 13. However, there is little information on the role of ANE in HCC therapy.

In the current study, we demonstrated that the ANE can inhibit the proliferation of HCC cells *in vitro* and *in vivo* through the activation autophagy and the induction of cell apoptosis. ANE-treated mice show little side effects. In conclusion, ANE may possess an anticancer effect against HCC, indicating that ANE may be a potential candidate for HCC therapy.

## Materials and methods

### Chemicals, reagents, and cell culture

Chemicals used in this study were obtained from Sigma (St. Louis, MO, USA). ANE compounds was provided by Dr. Tsung-Yun Liu and the preparation of ANE from ripe areca nuts was performed using protocols previously described 14. Antibodies targeting ATG12 (#4180), Beclin-1 (#3738), LC3 (#2775), Bcl-2 (#2872), and Bax (#2772) were from Cell Signaling Technology (Danvers, MA, USA), and antibodies targeting GAPDH (sc-47724), and cleaved poly (ADP ribose) polymerase (c-PARP) (sc-7150) were purchased from Santa Cruz Biotechnology (Santa Cruz, CA, USA). HepG2 cells were purchased from American Type Culture Collection (ATCC, Manassas, VA, USA), and Mahlavu and HepJ5 cells were established by Dr. C.S. Yang as previously described 15. HCC cell lines (HepG2, HepJ5, and Mahlavu) were grown in Dulbecco's modified Eagle's medium (Life Technologies, Grand Island, NY, USA) supplemented with 10 % (v/v) fetal calf serum in a 5% CO_2_ humidified incubator at 37 °C.

### Sulforhodamine B (SRB) colorimetric assay for cytotoxicity screening

Cells (2×10^4^) were seeded in 24-well plates and incubated overnight, followed by the addition of different doses of ANE (0~50 μg/ml) or vehicle for 48 h. Then, cells were fixed with 10% trichloroacetic acid for an hour fixation at 4 °C and stained with protein-bound SRB for 30 min. Next, the excess dye was removed, and cells were washed twice with 1% acetic acid. The dye was dissolved in 10 mM Tris base solution for OD measurements at 515 nm using a microplate reader (Bio-Rad Laboratories, Hercules, CA, USA) 16.

### Terminal deoxynucleotidyl transferase-mediated nick end labeling (TUNEL) assay

Cells were plated in six-well plates at 2.4×10^5^ cells/well overnight and then treated with 30 μg/mL ANE or H_2_O as the vehicle control for 48 h. Cells were harvested and washed with PBS. The cellular DNA fragmentation morphology was detected by a TUNEL assay using an Apo-BrdU *in situ* DNA Fragmentation Assay Kit (Bio Vision, Mountain View, CA, USA) according to the manufacturer's instructions. TUNEL-positive cells were then analyzed using flow cytometry.

### Total reactive oxygen species (ROS)/superoxide detection

ROS were measured using a total ROS/Superoxide Detection Kit (Enzo Life Science, Farmingdale, NY, USA) according to the manufacturer's instructions. Cells were stained with the two-color ROS Detection Kit and analyzed using a NucleoCounter^®^ NC-3000^TM^ system (ChemoMetec, Allerod, Denmark). In brief, cells (2.4×10^5^) were seeded in six-well plates overnight and then exposed to the ANE or the vehicle for 24 hours. Cells were harvested, and ROS and oxidative stress were detected by staining with the two fluorescent dyes from the ROS-ID^®^ Total ROS/Superoxide detection kit (ENZ-51010, Enzo). In addition, harvested cells were stained with Hoechst-33342, which was used to detect the total cell population 17.

### Autophagy detection by acridine orange (AO) staining and an autophagy detection kit

#### AO staining

Briefly, 2.4×10^5^ HCC cells/well were seeded into a six-well plate and cultured overnight. Cells were treated with 30 µg/ml ANE for 24 h. Treated cells were subsequently harvested and stained with AO (100 µg/ml) for 15 min in the dark. Any morphological changes to nuclei were observed using fluorescence microscopy (IX-71, Olympus, Tokyo, Japan).

#### Autophagy detection by an autophagy detection kit

Autophagy was measured using a CYTO-ID® Autophagy Detection Kit (NZ-51031, Enzo), according to the manufacturer's instructions. Briefly, cells (2.4×10^5^) were seeded in six-well plates overnight and then treated with the ANE or vehicle for 24 h. Cells were then harvested and stained with fluorescent dyes to measure autophagic vacuoles and monitor the autophagic flux in lysosomally inhibited live cells using a florescence dye that selectively labels accumulated autophagic vacuoles and stains autophagic vacuoles, including pre-autophagosomes, autophagosomes, and autolysosomes. The florescence intensity and number were detected and measured using the NucleoCounter^®^ NC-3000^TM^ system (ChemoMetec, Allerod, Denmark).

### Lysosome formation detection

ANE-induced lysosome formation was measured by a LYSO-ID® Green detection kit (ENZ-51034, Enzo). The dye accumulates in acidic compartments, such as endosomes, lysosomes, and secretory vesicles. Briefly, cells (2.4×10^5^) were seeded in six-well plates overnight and then treated with the ANE or vehicle for 48 h. Cells were then harvested and stained with fluorescent dyes from a LYSO-ID® Green detection kit and measured using the NucleoCounter® NC-3000^TM^ system (ChemoMetec).

### Protein extraction and Western blot analysis

Cells were treated with the ANE or vehicle for 48 h. Cell pellets were collected and were lysed by lysis buffer containing M-PER reagent, phosphatase and protease inhibitor (Boehringer Mannheim, Indianapolis, IN). Proteins were analyzed by Western blotting, as previously described^18^. In brief, proteins (20 μg) were separated by sodium dodecylsulfate polyacrylamide gel electrophoresis (SDS-PAGE), and electrotransferred onto polyvinylidene difluoride membranes (GE Healthcare Piscataway, NJ, USA). Membranes were incubated with ATG12 (1:1000), Beclin-1 (1:2000), LC3 (1:2000), Bcl2 (1:5000), Bax (1:2000), or c-PARP (1:2000) antibodies at 4 °C overnight, and subsequently probed with the respective secondary antibody. The products were visualized with an enhanced chemiluminescence reagent (GE Healthcare Piscataway, NJ, USA), and detected using VersaDoc 5000 (Bio-Rad Laboratories, Hercules, CA, USA).

### *In vivo* tumor xenograft experiments

All mouse experiments were performed in strict accordance with regulations of the Institutional Animal Care and Use Committee (IACUC), Taipei Medical University. Male NU/NU mice (5 weeks old) were used for the *in vivo* experimental model. HepJ5 cells were suspended in PBS to a final cell density of 10^7^ cells/ml. A volume of 0.1 ml of the cell suspension was injected subcutaneously (s.c.) into left side of each mouse. After 2 weeks, we began ANE treatment (20 mg/kg) twice per week by an intraperitoneal injection. Tumor dimensions and body weights were recorded twice per week. Tumor volumes were calculated using the equation (L×w^2^)/2, where L and w are the larger and smaller tumor dimensions, respectively 19. After 3 weeks, the mice were sacrificed, and all tumors were excised and weighed. Half of the excised tumor tissue was fixed in 10% formalin and embedded in paraffin for immunohistochemical staining; the other half was snap-frozen in liquid nitrogen for further evaluation.

### *In vivo* system to check the carcinogenesis effect of ANE

Male NU/NU mice (5 weeks old) were used for the *in vivo* experimental model. We began ANE treatment (40 mg/kg) twice per week by an intraperitoneal injection. After 4 weeks' treatment, the mice were performed PET scan analysis before sacrificed.

### PET analysis

All PET image data were processed by using the PMOD PNEURO software tool (version 3.7, PMOD Technologies Ltd., Zürich, Switzerland). All images were visually checked for correct coregistration and appropriate segmentation. For each PET scan image, a liver contour was generated using mannual standarized-uptake-value (SUV). From the static PET-data, SUVmean and SUVmax within the liver were calculated. For the dynamic PET-CT scans, a tumor contour was obtained from the last time frame of the dynamic PET-scan using PMOD.

### Statistical analysis

Data are presented as the mean ± standard deviation (SD) of at least three independent experiments. Significant differences were analyzed using Student's *t*-test (two-tailed) to compare two groups with *p* < 0.05 considered statistically significant.

## Results

### The ANE selectively inhibits the growth and induces cell apoptosis on HCC cells

**Figure 1:**
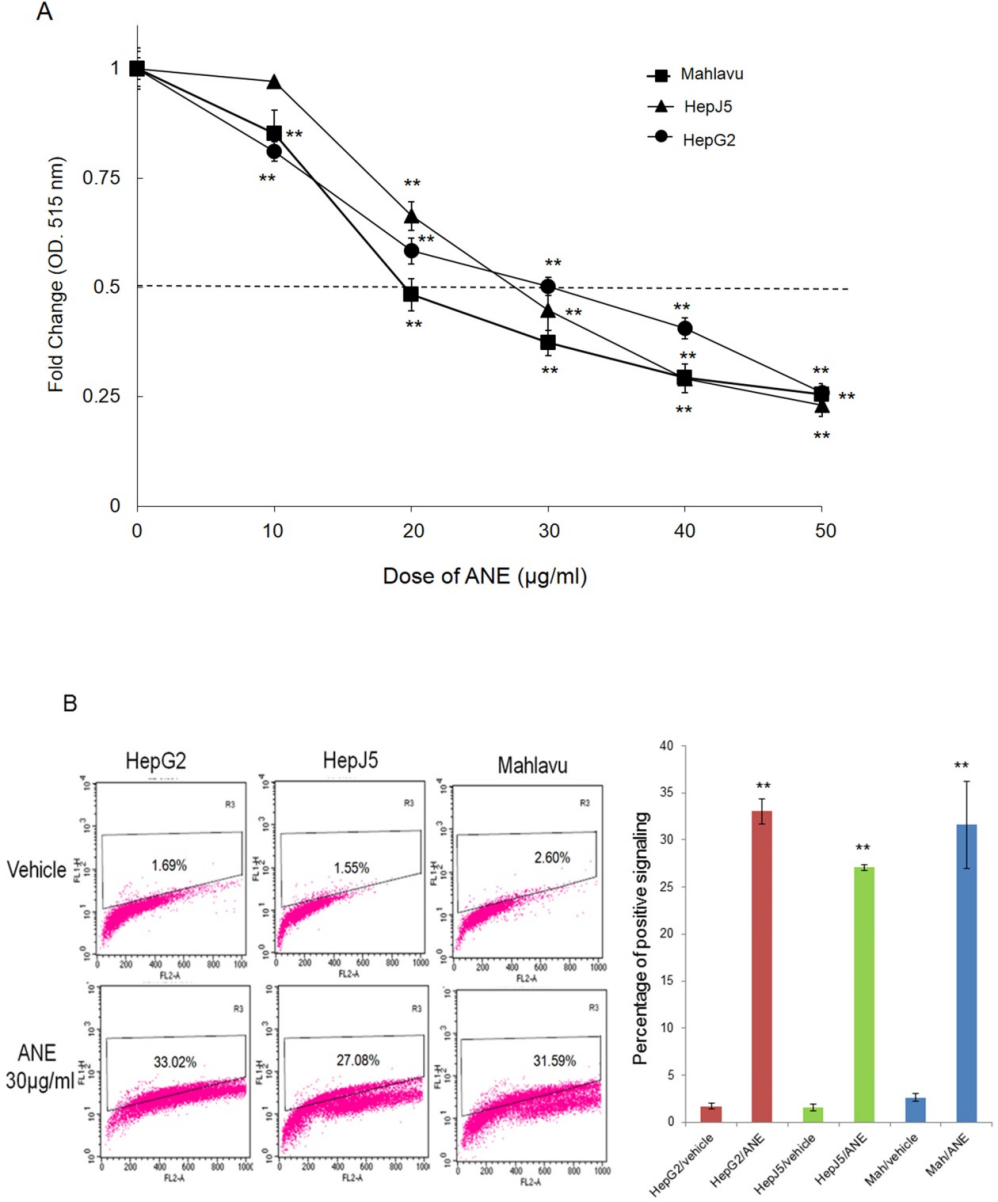
** ANE treatment decreases the cell survival rate and induction of apoptosis in HCC cells. (A)** HCC cells were incubated with different dose of ANE (0-50 µg/ml) for 48h. Cell survival rates were determined by SRB. Relative survival rate was set the vehicle treatment to be 100%. **(B)** HCC cells were treated with 30 ug/ml ANE for 48h. The apoptosis of HCC cells was determined by the TUNEL assay. The bar graph represents the percentage of positive signaling in TUNEL assay. Percentage of positive signaling of ANE treated cells was significantly increased as compared to the vehicle control. Data means ±SD of three independent experiments in triplicates (** p<0.01).

In order to check the effect of the ANE on HCC cells, we treated HepG2, HepJ5, and Mahlavu cells with different amounts of the ANE, and cell survival rates were determined. As demonstrated in Fig. 1A, cell survival rates of HepG2, HepJ5, and Mahlavu cells markedly decreased following ANE treatment in dose-dependent manners. The 50% inhibitory concentration (IC_50_) of the ANE was around 20~30 µg/ml after 48 h of treatment. This indicated that the ANE effectively inhibited the proliferation of HepG2, HepJ5, and Mahlavu cells.

### TUNEL assay

To further confirm that ANE treatment induced apoptosis of HCC cells, we performed a TUNEL assay. As shown in Fig. 1B, positive signals were < 5% in vehicle-treated HepG2, HepJ5 and Mahlavu cells. However, positive signals were increased to around 30% in ANE-treated HepG2, HepJ5 and Mahlavu cells (Fig. 1B). These results indicate that the ANE induced a growth-inhibitory effect through the induction of cell apoptosis.

### ANE treatment induces ROS generation

**Figure 2:**
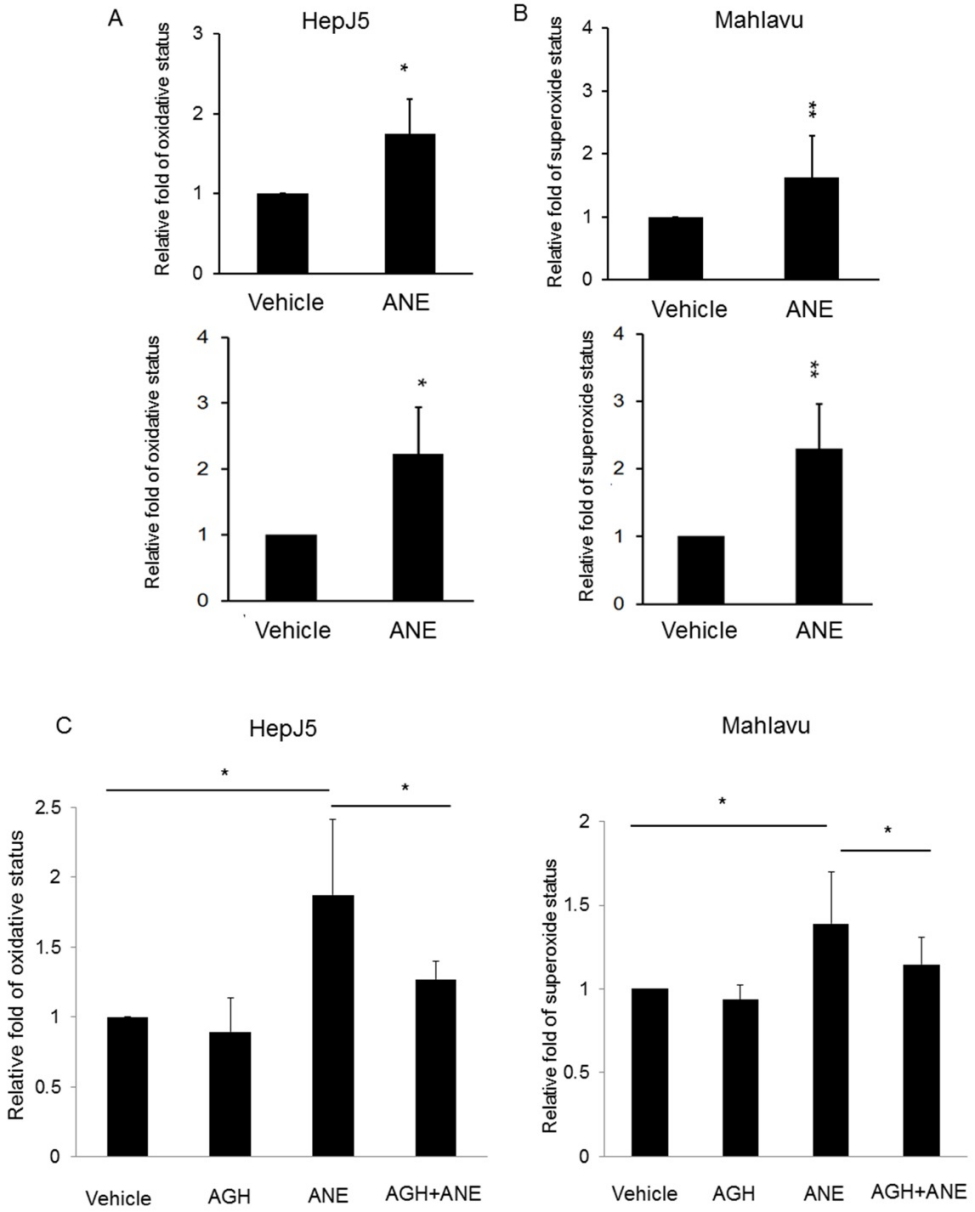
** ANE exposure induces ROS production and reversed by pre-treated antioxidant reagent in HCC cells.** HepJ5 and Mahlavu cells were treated with 30 ug/ml ANE for 24h. Oxidative and superoxide production was determined by ROS/Superoxide detection kit. **(A)** The relative ROS production were set by the increased fold compared with vehicle-treated samples. The ROS amount was increased approximately 2-fold in ANE-treated HepJ5 and Mahlavu cells compared to vehicle. **(B)** The relative superoxide amount was represented by the fold change compared with vehicle treatment. The superoxide status was increased by 2.3-fold after ANE-exposed on HepJ5 and Mahlavu cells. **(C)** ANE-induced ROS and superoxide formation was abolished after pretreatment with an antioxidant compound. Data means ±SD of three independent experiments in triplicates (* p<0.05, ** p<0.01).

ROS were observed to be involved in the induction of apoptosis in a number of systems. In order to determine whether ROS production was involved in ANE-induced apoptosis of HCC cells, relative ROS and reactive nitrogen species production was calculated in ANE-treated HepJ5 and Mahlavu cells using an ROS-ID® Total ROS/Superoxide detection kit. As shown in Fig. 2A, the ROS amount increased by approximately 2-fold in ANE-treated HepJ5 and Mahlavu cells compared to vehicle-treated cells. Superoxide was induced by 2.3-fold after ANE exposure of HepJ5 and Mahlavu cells (Fig. 2B). These results indicated that treatment with the ANE elevated intracellular ROS levels in HepJ5 and Mahlavu cells.

### An ROS blocker reverses ANE-induced ROS generation

To confirm the results obtained above, we explored if inhibition of oxidative stress with the antioxidant aminoguanidine hemisulfate (AGH) would affect the role of the ANE on inducing ROS/RNS and superoxide. As shown in Fig. 2C, we found that the ROS level was similar to the vehicle-treated HepJ5 and Mahlavu cells. ROS production dramatically increased in ANE-treated HepJ5 and Mahlavu cells. However, pretreatment with AGH with subsequent ANE exposure induced fewer ROS compared with ANE-treated only cells (Fig. 2C). These results indicated that ROS induction by ANE treatment could be reversed by antioxidant compounds.

### ANE treatment induces activation of autophagy and lysosome formation

**Figure 3:**
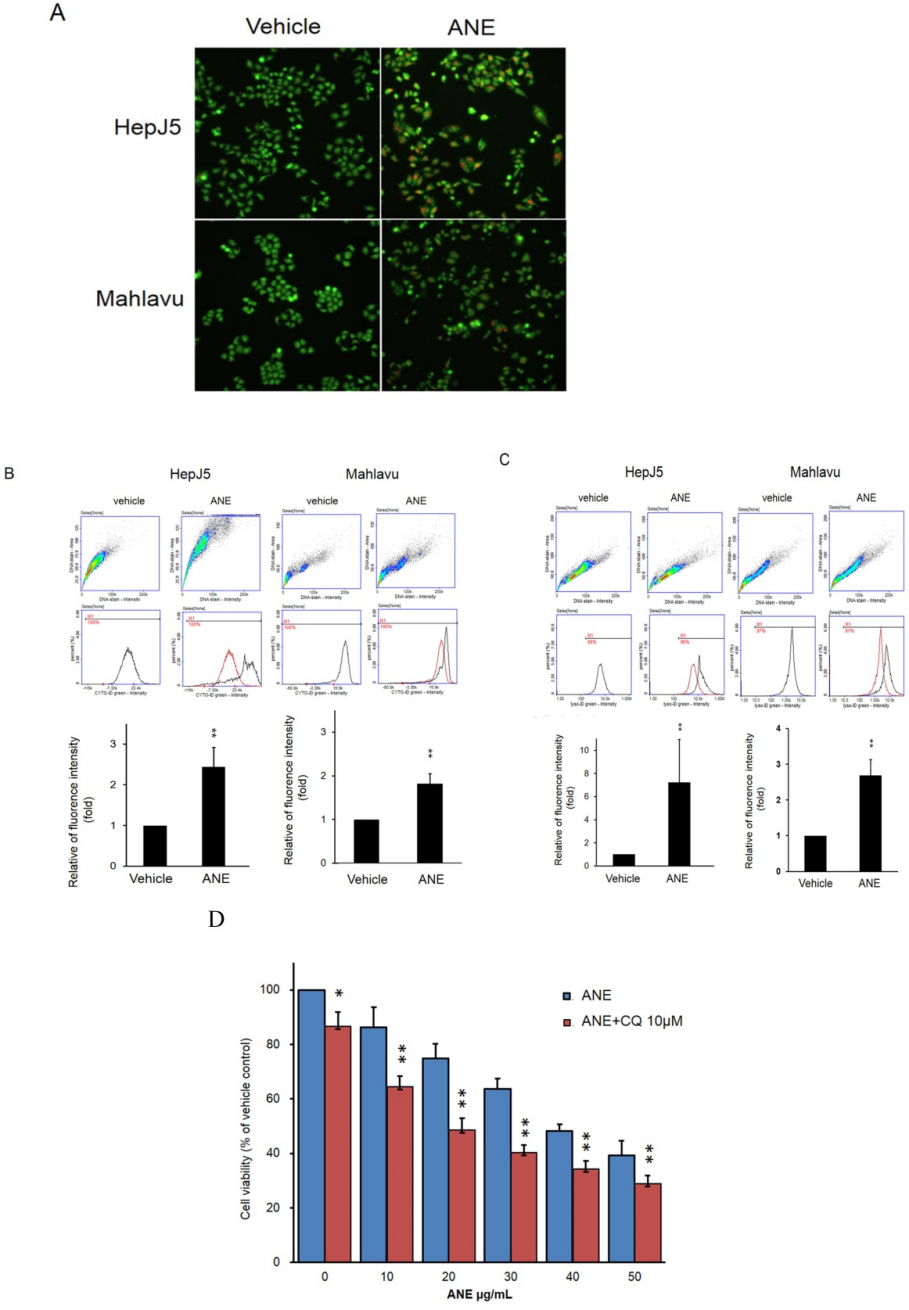
** ANE causes activation of autophagy and lysosome formation in HCC cells.** The autophagy activation by ANE were detected by acridine orange (AO) staining and Autophagy Detection Kit.** (A)** HepJ5 and Mahlavu cells were treated with ANE for 24 hr. The cells were stained by acridine orange. Positive signals were increased dramatically in ANE-treated HepJ5 and Mahlavu cells groups. **(B)** Fluorescence intensity was increased in ANE-treated HepJ5 and Mahlavu cells as compared to the vehicle. **(C)** The lysosome formation by ANE were detected by LYSO-ID® Green detection kit. HepJ5 and Mahlavu cells were treated with ANE for 48 hr. Fluorescence intensity was used to detect the formation of lysosome. Amount of lysosome was increased in ANE treated HepJ5 and Mahlavu cells. Data means ±SD of three independent experiments in triplicates (** p<0.01). **(D)** Blocking autophagy enhances ANE-induced cytotoxicity in HepJ5 cells. HepJ5 cells were treated with 0-50 µg/ml with or without 10 µM CQ. The cell viability in cells treated with ANE in the presence and absence of CQ was determined using SRB assay.

Autophagy plays a key role in maintaining cell growth in cancer progression. We further checked the effect of the ANE in autophagy activation. The present study detected autophagy activation in ANE-treated HepJ5 and Mahlavu cells using AO staining and a CYTO-ID® Autophagy Detection Kit. As shown in Fig. 3A, there were few positive signals after AO staining in vehicle-treated cells. However, orange signals dramatically increased in ANE-treated groups (Fig. 3A). The induction of autophagy after ANE treatment was confirmed using a CYTO-ID® Autophagy Detection Kit. As shown in Fig. 3B, positive signals increased by over 2~2.5-fold in ANE-treated HepJ5 and Mahlavu cells. We further checked lysosome formation using a LYSO-ID® Green detection kit. The formation of lysosomes was detected by the florescence intensity. As shown in Fig. 3C, ANE treatment increased the signaling of the amount of lysosomes in ANE-treated cells. These results indicate that ANE treatment may induce the activation of autophagy and increase the lysosome formation. To determine the role of autophagy in regulating MG-induced cell death in HCC, CQ (chloroquine, a lysosomal inhibitor) was used in ANE-treated HepJ5 cells. It was found that combinatorial treatment of CQ significantly increased ANE-induced cytotoxicity in HCC. As shown in Fig. 3D, the cell viability was decreased in ANE plus 10 µM CQ treatment compared to ANE only.

### ANE treatment suppressed HCC progression in a xenograft mouse model

**Figure 4:**
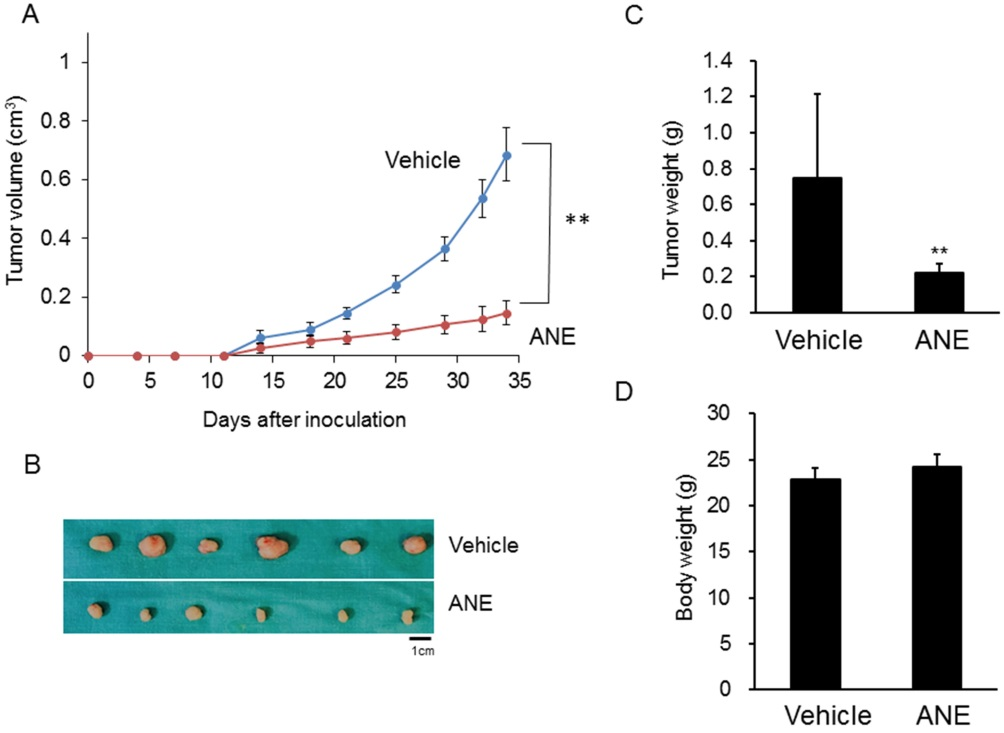
** ANE inhibits cancer progression in a xenograft mice model.** The xenograft model was applied to check the effect of ANE on HCC. 1× 10^6^ HepJ5 cells were injected into the left side flanks of nude mice. **(A)** The tumor volume was measured twice per week. **(B)** After 4 weeks' treatment, mice were sacrificed and the outlook for tumors were taken. The tumor sizes and growth rate in ANE-treated group was significantly decreased more than 50 % as compared to the vehicle-treated group. **(C)** The tumor weights of the ANE-treated group were reduced dramatically to 30% compared with vehicle control. **(D)** There was no significant difference in body weights between the vehicle and ANE-treated mice group. Data means ±SD of three independent experiments in triplicates (** p<0.01).

To further confirm the *in vitro* findings, a xenograft mouse model was used to evaluate the inhibitory effect of the ANE on HCC progression. Tumor sizes and growth rates in the ANE-treated group significantly decreased by > 50% compared to the vehicle-treated group (Fig. 4A, B). Tumor weights in the ANE-treated group dramatically decreased to 30% compared to the vehicle control (Fig. 4C). However, there was no significant difference in body weights between the vehicle- and ANE-treated mice groups (Fig. 4D). These results indicated that the ANE treatment may suppress the cancer progression *in vivo*.

### ANE influences autophagy or apoptosis-related proteins expression patterns

**Figure 5:**
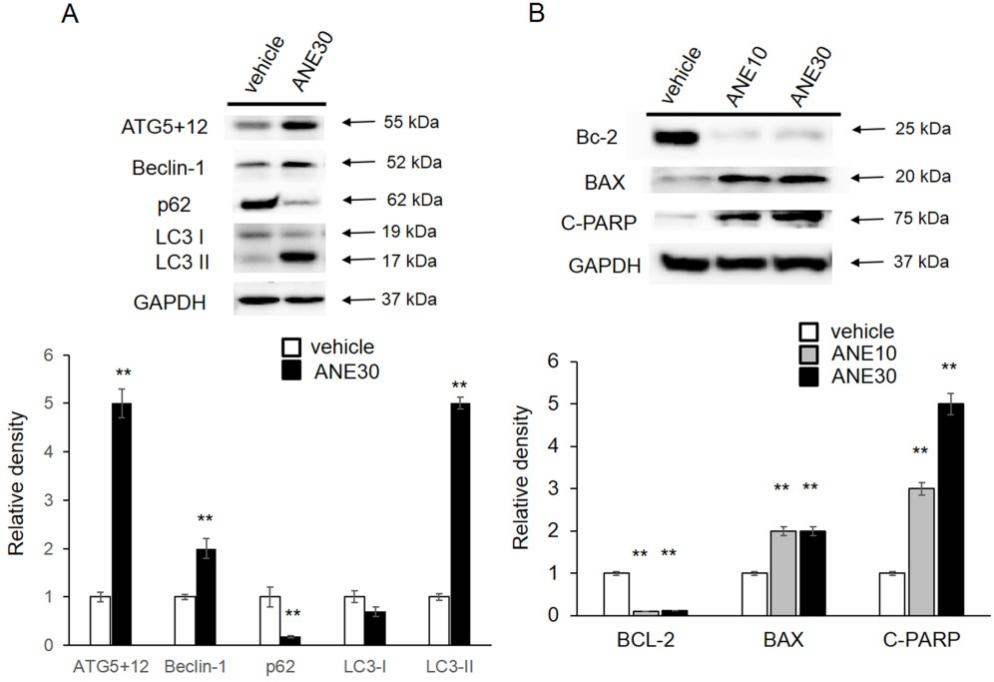
** ANE treatment causes changes in autophagy- and apoptosis-related proteins.** HepJ5 were treated with 30 ug/ml ANE or vehicle for 48 h. **(A)** The levels of autophagy related proteins (ATG5+12, Beclin-1, p62, and LC3) were checked by western blotting. The amount ofATG5+12, beclin-1 was increased in ANE-treated sample compared with vehicle control. The LC3-II was increase in ANE treated sample. **(B)** The cell apoptosis related proteins, Bcl-2, Bax, and c-PARP was checked. ANE treated cells showed decreased Bcl-2 (anti-apoptotic), increased Bax protein (pro-apoptotic) and increased cleavage PARP. All experiments were repeated at least three times independently (** p<0.01).

To further understanding the molecular mechanism, we checked the autophagy and apoptosis related protein levels by Western blotting. First, we checked levels the autophagy-related proteins (ATG5+12, Beclin-1, p62, and LC3-I/II), and results are shown in Fig. 5A. Amounts of LC3-II and ATG5+12, Beclin increased in ANE-treated samples compared to the vehicle control and a decrease of p62 after ANE exposure. Induction of those proteins was correlated with activation of the autophagic pathway. We further confirmed cell apoptosis-related proteins, including Bcl-2, Bax, and c-PARP. As shown in Fig. 5B, we found that ANE treatment caused a decrease in the Bcl-2 (antiapoptotic) and an increase in the Bax protein (proapoptotic). In addition, c-PARP increased after exposure to the ANE. Taken together, ANE treatment induced activation of autophagy and caused cell apoptosis.

### The effect of ANE treatment in health mice

**Figure 6:**
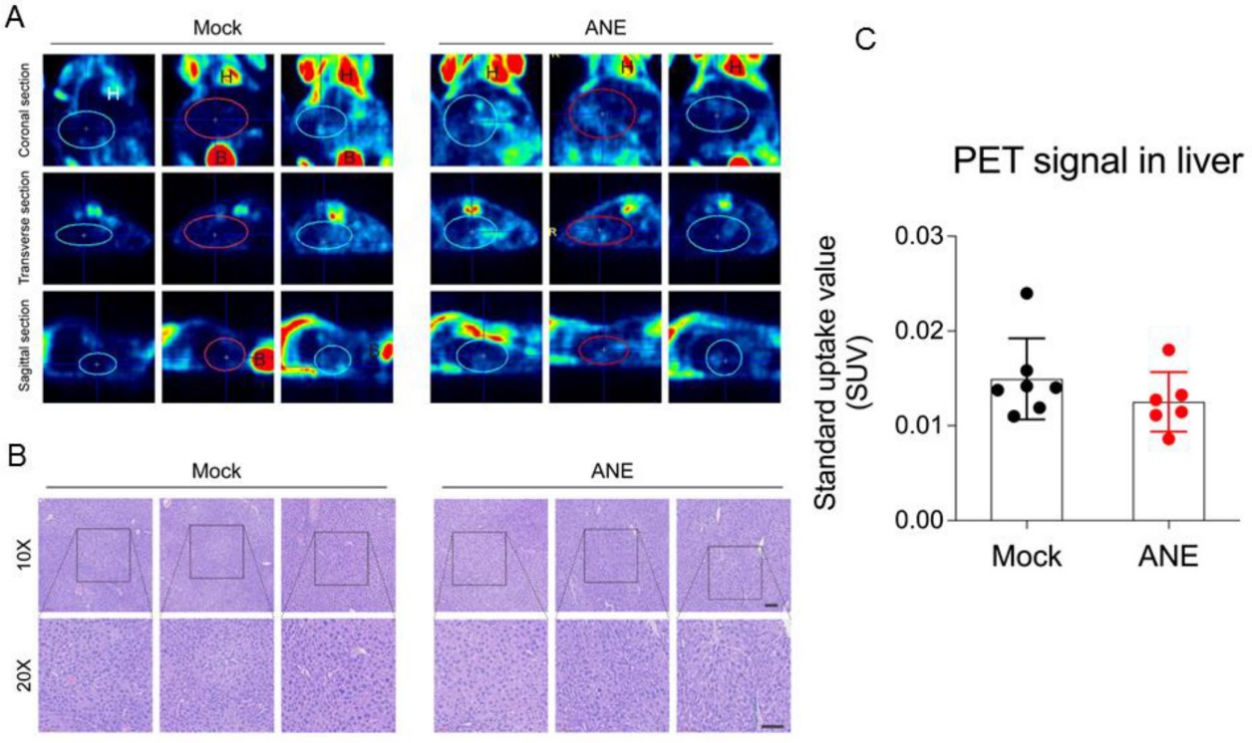
** ANE treatment has no significant tumorigenicity effect in immune competent mice. A.** Representative 18F-FDG-PET scaning imaging of whole mice by different sections. Blue/red circles indicate the liver region. H: heart; B: bladder. **B.** Representative hematoxylin & eosin (H&E) stained images of liver tissues from mice following long-term low-dose ANE treatment. Scale bar: 100 µm. **C.** Statistic bar graph of standarized-uptake-value (SUV) from A. *p* value was calculated by unpaired *t* test. Experiments were performed in three biological replicates and presented as mean ± standard errors. Mice number are 8 and 7 in mock and ANE group, respectively.

To understand the tumorigenecity effect of long-term low-dose ANE treatment in *in vivo* animal model, nude mice were intraperitoneal injected with water (control) or ANE (40 mg/kg) twice per week for 30 days. Mice were performed 18F-FDG-PET scanning in day 30 post-injection. 18F-FDG-PET scanning image revealed no significant FDG uptakes signal in the liver (SUVmean 0.01403+0.00428) of mock control mice (Fig. 6A and C). Furthermore, liver tissue from mice with long-term drug treatment was analyzed by histopathological examination. Data from Fig. 6B revealed no significant differences between mock and ANE group. Meanwhile, we also observed other organ from ANE-treated mice, including heart, lung and kidney (data not shown); there were no significant changes in histopathological examination.

## Discussion

Improving the clinical management of HCC is of paramount importance. Although the management of HCC has remarkably improved in recent years, high relapse rates and high toxicities of current modalities hinder their clinical success. In order to check the anticancer effect of the ANE, we treated HCC cells with the ANE and found an inhibitory ability of the ANE against HCC cells *in vitro* (Fig. 1) and *in vivo* (Fig. 4). To realize the carcinogenesis effect of ANE, higher dose ANE (40 mg/kg) was injected to mice for 4 weeks and found no significant differences between mock and ANE group (Fig. 6). These results indicated that the ANE may be a potential compound for HCC therapy.

The ANE is inexpensive and easily accessed, and was demonstrated to have few side effects when used to treat different diseases 5, and our results agree with those findings as little toxicity was demonstrated in mice. Another advantage is that it is locally available and well known as a medicinal plant with a wide spectrum of biological and pharmacological activities, like antioxidative and anti-inflammatory properties, including treatment of liver disorders 20. This is a big advantage, as the success of many anticancer agents is hindered by their high price and high toxicity. Therefore, ANE may offer an affordable alternative for treating HCC.

The ANE is well studied for its role in inducing oral cancer 21-23. It was also demonstrated to be cytotoxic to normal oral epithelial cells, while adjacent fibroblasts, which are more resistant, result in induction of oral submucosal fibrosis (OSF), a premalignant and fibrosing disease that may lead to cancer 6, 24-26. However, in cholangiocarcinoma (CCA) cells, the ANE exhibited antiproliferative effects even at lower doses and dramatically increased apoptotic signaling, by modulating apoptotic molecules like caspase-3 and -7 and c-PARP 27. This agrees with our study and gives insights into the other role of the ANE in cancers, as induction of apoptotic signaling by ANE treatment was also found, as evidenced by an increase in c-PARP and a decrease in the Bcl-2 molecule (Fig. 5B).

It was demonstrated to induce autophagy in both normal and cancerous cells through oxidative stress 26, 28, 29. Many anticancer agents cause metabolic stress to cancer cells, and this results in increased ROS concentrations 30-32. Oxidative stress in cells causes intracellular accumulation of misfolded proteins, which induces autophagy 33-36. The ANE was shown to increase ROS (Fig. 2), while at the same time promoting both autophagy, as evidenced by the reduction in the LC3I/II ratio, p62 degradation, and increases in levels of autophagy proteins (ATG5+12 and Beclin-1) suggesting that the ANE induced HCC cytotoxic effects through ROS signaling in this study (Fig. 5). The production of ROS as a result of loss of the mitochondrial membrane potential is the proposed mechanism said to promote both apoptotic and autophagic cell death 37, 38. While providing insights into possible interactions between apoptosis and autophagy, this study showed that the ANE is a possible anticancer agent for treating HCC. However, the major concern may need to further understand what's the major anticancer component within ANE because ANE is considered to be a carcinogen. Even though, we didn't find the damage in major organ after four-week ANE treatment in mice. It still can't make sure the safety for clinical application. That's the limitation for this study. To understand the functional anticancer compounds within ANE, it may be the possible direction to develop. In conclusion, our results imply that ANE may be a possible therapeutic strategy for HCC.

## Significance of this study

***The current landscape:***

- HCC is the third leading cause of all cancer-related deaths worldwide;
- The therapeutic outcome in HCC patients is not satisfied;
- Areca nut extract (ANE) may be correlated to the oral cancer through the carcinogenesis in oral epithelial cells.

***New findings:***

- ANE treatment contains the anti-HCC effect *in vitro* and *in vivo* xenograft model;
- Four weeks ip. injection ANE did not see the carcinogenesis effect in mouse model;
- ANE treatment induced the ROS production and induced apoptosis.

***Clinical significance and impact:***

- Our study provides clues that ANE may contain the anti-HCC effect;
- The compounds in ANE may be the potential anti-cancer component in clinical.

## Figures and Tables

**Figure 7:**
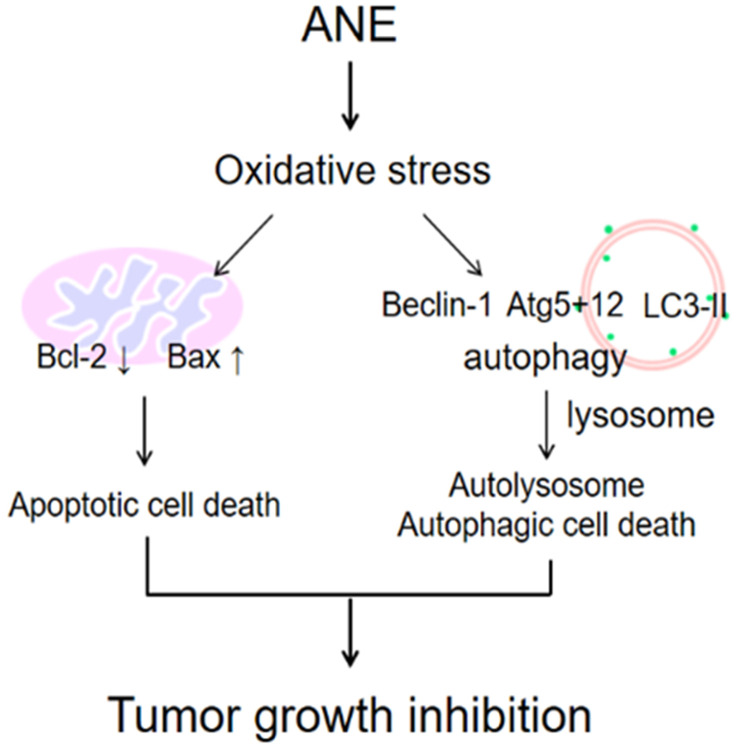
The schematic plot to summery the therapeutic effect of ANE in HCC.

## Abbreviations

HCC: Hepatocellular carcinoma
ANE: areca nut extract
AGH: aminoguanidine hemisulfate
ATG: anti-thymocyte globulin
ROS: Reactive oxygen species
